# Links between learning goals, learning activities, and learning outcomes in simulation-based clinical skills training: a systematic review of the veterinary literature

**DOI:** 10.3389/fvets.2024.1463642

**Published:** 2024-10-02

**Authors:** Neeltje J. Veenema, Beerend P. Hierck, Harold G. J. Bok, Daniela C. F. Salvatori

**Affiliations:** ^1^Department of Clinical Sciences, Division of Anatomy and Physiology, Faculty of Veterinary Medicine, Utrecht University, Utrecht, Netherlands; ^2^Department of Population Health Sciences, Division of Farm Animal Health, Faculty of Veterinary Medicine, Utrecht University, Utrecht, Netherlands

**Keywords:** learning goals, learning activity, learning outcomes, clinical skills training, simulation-based education, experiential learning theory, constructive alignment

## Abstract

**Introduction:**

In veterinary education programs it is important to have a balance between providing students with valuable hands-on experience and ensuring the ethical treatment and welfare of the animals involved. In the last years simulation-based veterinary education played an important role helping with the replacement of experimental animals in education and at the same time creating a safe learning environment offering endless options for training in a safe environment. The aim of this systematic review was to discern which type of learning outcomes are used to evaluate specific learning goals of clinical skills training and to grasp the impact of diverse simulator characteristics on the measured learning outcomes in clinical skills training.

**Methods:**

A systematic search from 1977 until November 2023 has been conducted resulting in 103 included papers. The categories, learning goals, learning activities, and learning outcomes in clinical skills training were used for data extraction of all included studies.

**Results:**

This study investigated the interplay between learning goals, learning activities, and learning outcomes. Competence and knowledge were the most frequently described learning outcomes; static and screen-based simulators are the are most frequently used technologies. Static simulators are primarily used to train procedural steps and screen-based simulators are primarily used to train relevant knowledge and clinical reasoning. Notably, none of the reviewed studies made explicit connections between learning goals, learning activities, and learning outcomes.

**Discussion:**

In simulation-based education it is important to provide a structured, constructively aligned process where students gain relevant and effective experience. The results of this study underscore the importance of aligning the learning process in simulation-based clinical skills training, and that alignment in the learning process is not always evident.

## Introduction

Clinical training plays a vital role in preparing veterinary students for their professional careers. The primary objective of clinical training is to acquire the essential clinical skills required to deliver optimal care and treatment to animals. The acquisition of clinical skills can be facilitated through a range of learning modalities, spanning from traditional textbooks to mastery learning sessions in the clinic, as well as using both low and high-fidelity simulation models. In veterinary education, students commonly engage with experimental (non-patient) animals to acquire practical experience in various procedures, examinations, and treatments. However, it is crucial to recognize the potential impact on the welfare of these experimental animals (1). Certain procedures or treatments performed during veterinary education may cause discomfort to the animals, and because of this reason can be repeated only a limited number of times, potentially limiting exposure and training possibilities for students. It is important for veterinary education programs to strike a balance between providing students with valuable hands-on experience and ensuring the ethical treatment and welfare of the animals involved (1). This can be achieved through animal-free simulation-based veterinary education (SBVE). Many animal-free simulators have already been developed and organized in, e.g., skills labs (2, 3). Animal-free models are designed to align with ethical standards and the humane treatment of animals. The concept of animal-free models is rooted in the principles of the 3Rs, i.e., replacement, reduction, and refinement. These principles advocate for finding alternatives to animal use, minimizing the number of animals used, and refining procedures to reduce harm to animals (4, 5). This approach is particularly valuable for ensuring that students develop necessary clinical skills before working with live animals.

In addition, animal-free models can offer a level of standardization and reproducibility that may be challenging to achieve with live animals. This consistency enhances the learning process by allowing students to practice and repeat procedures under controlled conditions (6). SBVE aligns with the experiential learning theory (ELT) by providing a structured and deliberate environment for students to actively participate in clinical scenarios (7). Experiential learning theory is a framework that emphasizes the central role of experience in the learning process (8–10). The theory posits that learning is a cyclical and iterative process. The iterative nature of experiential learning in simulation-based education is reflected in the involvement of repeated cycles of simulation training, reflection, and application (8–10). This process allows students to progressively build and refine their clinical skills. Conducting training of clinical skills in a simulation-based environment will allow for mistakes and the repetition of complex procedures without inflicting harm to a living animal. Students can progress at their own speed, ensuring that each individual masters the skill before moving on to other (more complex) scenarios (1, 10–15). SBVE allows for further innovation and quick adaptations to state-of-the-art methods to enhance clinical skills training for the next generation of veterinary professionals.

SBVE has become the subject of increasing investigation and reporting and aiming to setup organized skills labs (2, 3, 12, 16, 17). Notably, the study by Noyes et al. suggests that simulator training within veterinary education can yield positive outcomes for clinical skills development (2). However, the effectiveness of SBVE is evaluated through diverse learning outcomes, including knowledge acquisition, retention, procedural skills acquisition, quality of clinical decision-making, and confidence levels. This variety in criteria complicates the interpretation and comparison of SBVE effectiveness across studies (2). Moreover, while there is existing research exploring the general effectiveness of SBVE and the influence of simulator characteristics (e.g., fidelity) on overall effectiveness, there is limited literature examining how specific simulator characteristics impact individual, separate learning outcomes. This gap in research highlights the need for a comprehensive literature review to elucidate the relationship between simulator characteristics and individual learning outcomes in veterinary clinical skills training. Given the relative lack of exploration in this area, a systematic review is warranted to address this gap and to provide insights into optimizing SBVE for enhanced educational outcomes in veterinary medicine.

A clinical skill is a comprehensive and all-encompassing concept that involves various components open to training and refinement. The clinical skills training consists of a learning process that entails interrelated components, specifically, learning goals, learning activities, and learning outcomes. Learning goals are the specific objectives that define what students are expected to know or achieve and lend themselves to focused training. Learning activities are the practical experiences, simulations, or exercises designed to facilitate the attainment of these goals. Learning outcomes are the results of clinical skill training. Within a specific clinical skills training, one or multiple learning outcomes can be measured and reported, providing a nuanced evaluation of both the acquired (sub) skills through that training and prior initial (sub)skills (18, 19). In the scope of this review the focus is on these separate measurable learning outcomes. Moreover, it is important to recognize that the results of clinical skills training can extend beyond the formal learning objectives, e.g., boosting self-efficacy or social skills. Together, learning goals, learning activities, and learning outcomes create the scaffolds of a learning process for students to develop the practical abilities required in clinical settings (2, 18–20). Investigating how specific simulator characteristics contribute to the learning process of students during simulation-based clinical skills training can provide new insights. In essence, the purpose of this systematic review is to discern which type of learning outcomes are used to evaluate specific learning goals of clinical skills training and to grasp the impact of diverse simulator characteristics on the measured learning outcomes in clinical skills training.

To address the aforementioned aim, the relation between learning outcomes and simulation-based clinical skills training in veterinary education was investigated.

In particular we addressed:

1) How do the Learning outcomes relate to the specific Learning goals during clinical skills training?
2) How do simulator characteristics in the Learning activity relate to the measured Learning outcomes in the clinical skills training setting?

## Methods

This systematic review was conducted according to the Preferred Reporting Items for Systematic Reviews and Meta-Analyses (PRISMA) standards of quality (21). This study has been preregistered on Open Science Framework (osf.io/3m6fz).

### Search strategy

A systematic search in relevant literature published from 1977 until November 2023 was performed using the following electronic databases: Scopus, MEDLINE (PubMed), Embase, ERIC and CAB Abstracts. The search consisted of the subject terms and subsequent Boolean combinations in each database for the terms “education” AND “model OR simulation” AND “veterinary” AND “outcomes” (see Supplementary Document 1).

A manual search was also performed to retrieve additional studies for this review. The manual search consisted of reviewing the reference lists of identified papers and relevant systematic reviews to find additional relevant studies. Duplicates were identified through the reference program EndNote (Endnote 20, Clarivate) and removed from the total number of records identified through the initial search and the manual search.

### Study selection

**Figure 1:**
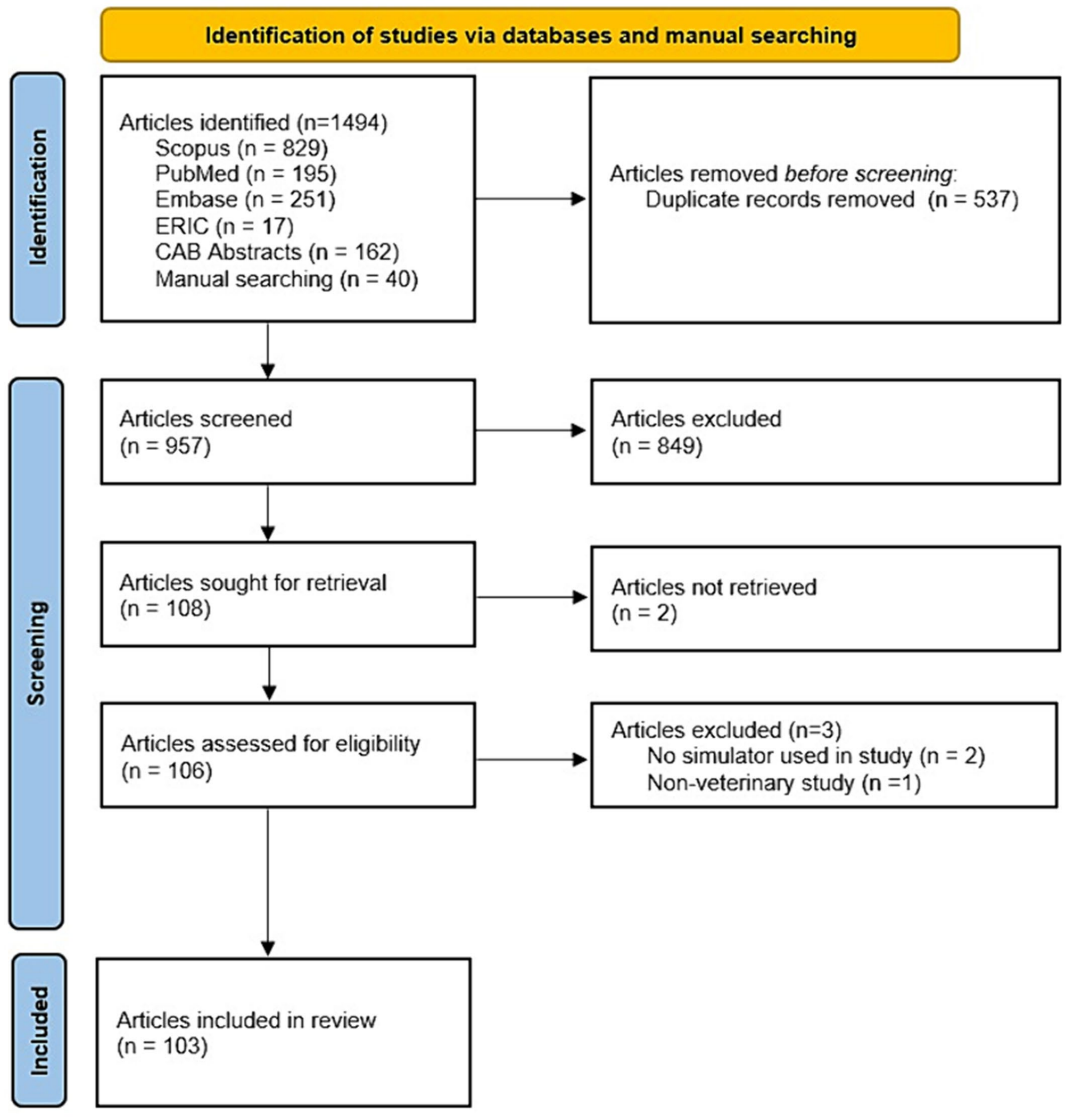
PRSIMA flowchart of search strategy.

The remaining records were screened by reviewing the title and abstract. After the initial title and abstract-based review process, all retrieved full-text papers were assessed for eligibility based on the in-and exclusion criteria. Inclusion criteria were description of peer reviewed and original research; simulators used for skills training in veterinary education; veterinary students as studied population; skills had to be clinical skills; quantitative and/or qualitative measurements of cognitive and/or clinical skill outcomes; presence of a control group for comparison; full-text availability in English. All papers were tested against the in-and exclusion criteria by an AI method (ASReview) (22) and two independent reviewers, and disparity between reviewers for the inclusion of 27 papers was settled through consensus. This resulted in 103 papers that met all inclusion criteria and were included for the systematic review; see Figure 1. The risk of bias was assessed by one reviewer and checked by the other reviewers in the included studies using the revised Cochrane ‘Risk of bias’ tool (23).

### Data extraction and analysis

A data extraction table was created in Excel (Excel 2016, Microsoft) to capture study characteristics for the included studies (see Supplementary Document 2). In order to analyze the selected studies, three categories were identified: (1) “Learning goals,” (2) “Learning outcomes,” and (3) “Learning activities.” For each reviewed study, the definitions of these categories were applied to the extracted data. This means that, even if the reviewed studies did not use the same terminology or explicitly define their learning goals, learning activities, or learning outcomes in those terms, the reviewers interpreted the study data to fit into these predefined categories. This approach allowed for a structured analysis that identified how these three elements intersect and relate to clinical skills training in veterinary education.

### Learning goals

Clinical skills encompass various components that can be trained and refined (24). For each clinical skill it was identified per study whether the training included one or more of the following “Learning goals”: “Relevant knowledge,” “Clinical reasoning,” and/or “Procedural steps” (Table 1) (25). Furthermore, for each study it was determined whether the focus primarily was on either of these. This was done based on the proportion of content per study dedicated to relevant knowledge, clinical reasoning, and procedural steps. The percentage reported for each learning goal component reflects the proportion of studies that identified that particular component as the primary learning goal. For example, if 17% of the studies focused on clinical reasoning as a primary learning goal, that percentage is relative to the total number of included studies (Table 2).

**Table 1:** List of terminology and definitions used in the paper with focus on the “Learning goals,” “Learning activities,” and “Learning outcomes.”

Schematic summary of definitions used in the systematic review	Definition
Prior (sub)skills	The initial level of prior knowledge, clinical reasoning skills, or competence that students possess in specific clinical (sub)skills before they undergo clinical skills training.
Learning goals	
Relevant knowledge	Knowledge acquired during clinical skills training. Being familiar with the theoretical basis and/or understanding of the theory needed for successful performance of a clinical skill.
Clinical reasoning	Clinical reasoning skills acquired during clinical skills training. A dynamic and iterative process that involves both analytical thinking and practical judgment.
Procedural steps	Competence acquired during clinical skills training. The technique used to perform a clinical activity; and/or relates to the sequence of performance within the skill.
Learning activities	
Simulator technology	Simulator technology is categorized in static simulators, box trainers, screen-based simulators, XR technology, integrated simulators, and animal-derived simulators. Static simulators: fixed scenarios frozen in time. Box trainers: often equipped with essential laparoscopic tools such as lenses, cameras, light sources, and monitors, provide hands-on practical experiences. Screen-based simulators: using computer screens for the projection of information and possible interactive elements. Extended Reality (XR): includes Augmented Reality (AR), Virtual Reality (VR), and Mixed Reality (MR), integrating virtual elements into real or simulated environments. Integrated simulators: link various learning goals of clinical skills training, Animal-derived simulators: biological materials sourced from animals, such as cadavers, organs, milk, and eggs.
Type of simulator	The context of the training related to the simulator. Part-task-driven simulator: training of a specific procedure or intervention. Event-driven simulator: training with a focus on clinical situations and case simulation. Hybrid simulator: training containing (part-)tasks within the context of a clinical situation.
Use of simulator	Interactivity in using the simulator. Interactivity is defined as a simulator requiring a user’s input and the simulator responding to a user’s input. The simulator can be defined as Interactive in use or as Non-interactive in use.
Learning outcomes	
Knowledge	Measurement of integrated knowledge. Both prior knowledge and relevant acquired knowledge during learning activity are included.
Application of knowledge	Measurement of the implementation of integrated knowledge in a clinical scenario; includes diagnostic reasoning; and/or aspects of clinical decision making; and/or understanding of the limitation of the skill and when not to use it; and/or a degree of interpretation of clinical findings.
Competence	Measurement of integrated competency. Students demonstrate a clinical skill with desired competency levels in an educational setting (appropriate for level).
Clinical performance	Measurement of clinical performance. Students apply clinical skills independently in a clinical setting (e.g., daily patient care).

**Table 2:** Summary of study components related to all selected studies.

Component	Subject	Percentage (%) of studies
Primary Learning goal per study	Relevant knowledge Clinical reasoning Procedural steps	18% 17% 64%
Learning outcome [Self-eva]^a^	Knowledge Application of knowledge Competence Clinical performance	32% [28%] 17% [17%] 58% [50%] 4% [18%]
Simulator technology	Static simulator Screen-based simulator Box trainer XR technology Integrated simulator Animal-derived simulator	49% 31% 12% 6% 6% 5%
Type of simulator	Part-task-driven Event-driven Hybrid simulation	64% 29% 7%
Use of simulator	Interactive Non-interactive	94% 9%

The percentages indicate the prevalence of each component within the respective studies. The percentages across components may exceed 100% due to the presence of multiple elements within a single study.

a The Self-evaluated Learning outcomes [Self-eva] are depicted between brackets per category of learning outcome.

In the learning process of clinical skills “Relevant knowledge,” “Clinical reasoning,” and/or “Procedural steps” do not need to be all present in a specific learning scenario. This is illustrated in the following examples. For learning the procedural steps in performing rectal palpation in cattle, the student needs relevant knowledge of bovine anatomy and knowledge of the technique for rectal palpation. Procedural steps include restraint of the cow, lubrication of the rectal glove, gentle insertion of the hand into the rectum, and systematic palpation of the reproductive structures. Training in these tasks can take place without clinical reasoning.

For learning clinical reasoning skills in a simulated scenario featuring a case of a dehydrated cow, the student needs relevant knowledge of bovine physiology. Clinical reasoning includes the ability to evaluate the extent of dehydration and consider possible underlying factors. Subsequently, the student can make an appropriate treatment strategy based on their assessment. It is not necessary to perform procedural steps in this virtual case.

### Learning activities

For this review the simulator characteristics are described as the “Learning activity.” Three characteristics for each simulation model have been identified: (1) “Simulator technology,” (2) “Type of simulator,” and (3) “Use of simulation” (Table 1).

1) Simulator technology included static simulators, box trainers, screen-based simulators, virtual or augmented reality, integrated simulators, and animal-derived simulators (see Table 1 for definitions) (2, 14, 26, 27).
2) Type of simulator was subdivided into part-task-driven simulator, event-driven simulator, and hybrid simulator. A certain type of simulator can be useful for mastering individual components of a larger skillset (part-task), for training with a primary focus on clinical situations and case simulation (event) or an integration of specific tasks or procedures within the broader context of a clinical situation (hybrid) (15).
3) Use of simulator was defined as the simulator being interactive or non-interactive during use. Interactivity refers to the dynamic relationship between the user (student) and the simulator. It is defined by the extent to which the simulator requires input from the user and responds to that input. For example, in surgery simulators, interactivity may involve the user manipulating virtual surgical instruments, making incisions, and observing realistic responses such as bleeding or tissue reactions. Conversely, a simulator can be categorized as non-interactive when it operates in a more predetermined manner, with limited or no responsiveness to the user’s actions, like a screen-based simulator where the simulator only requires the student to watch a video without any input (28).

The prevalence of each subcategory was calculated by counting how many times it was identified across all reviewed studies. For instance, if within “Simulator technology” the static simulator was identified in 49% of the studies, this percentage reflects its occurrence relative to the total number of studies reviewed. As studies could involve multiple learning activities and subcategories, the combined percentages of these subcategories could exceed 100% (Table 2).

### Learning outcomes

Learning outcomes were defined as the results of a specific clinical skill training, in which one or multiple separate learning outcomes can be evaluated, measured, and given feedback on. The learning outcomes are an integration of both prior knowledge and (sub) skills and newly acquired knowledge and (sub) skills during the clinical skill training. It is important to mention that in this review a distinction is made between these separate measurable learning outcomes and formal assessments, where the latter are not included in this review. It was anticipated that individual studies would report data on multiple learning outcomes. To address possible preferences for some learning outcomes to be evaluated by self-evaluation (only), for each study the separate “Learning outcomes” were extracted and then divided into two categories: “Evaluated learning outcomes” and “Self-evaluated learning outcomes.” Both groups of learning outcomes were defined according to Miller’s pyramid in four distinctive types: “Knowledge,” “Application of knowledge,” “Competence,” and “Clinical performance” (Table 1) (29, 30). The percentages for each learning outcome component represent how often each outcome was observed relative to the total number of studies. Since multiple learning outcomes could be identified within a single study, the cumulative percentages could exceed 100%. For example, knowledge was found in 32% of studies, application of knowledge in 17%, and competence in 58%, resulting in a total more than 100% (Table 2).

**Figure 2:**
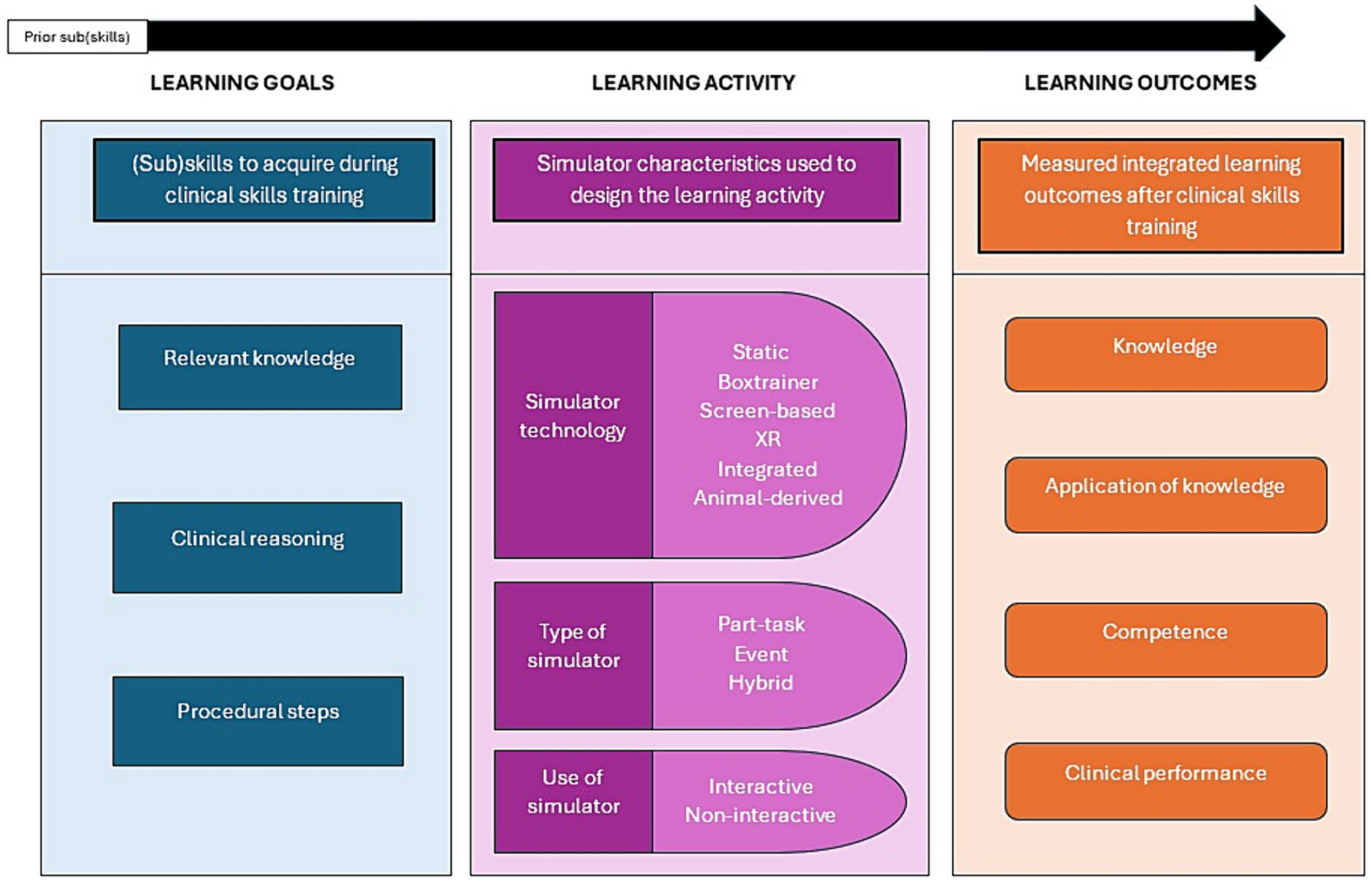
The categories, “Learning goals,” “Learning activities,” and “Learning outcomes” in clinical skills training as used for data extraction.

The aim was to examine how the learning outcomes relate to the specific learning goals during clinical skills training and how simulator characteristics in the learning activity relate to the learning goals and measured learning outcomes in the clinical skills training setting (Figure 2).

## Results

**Figure 3:**
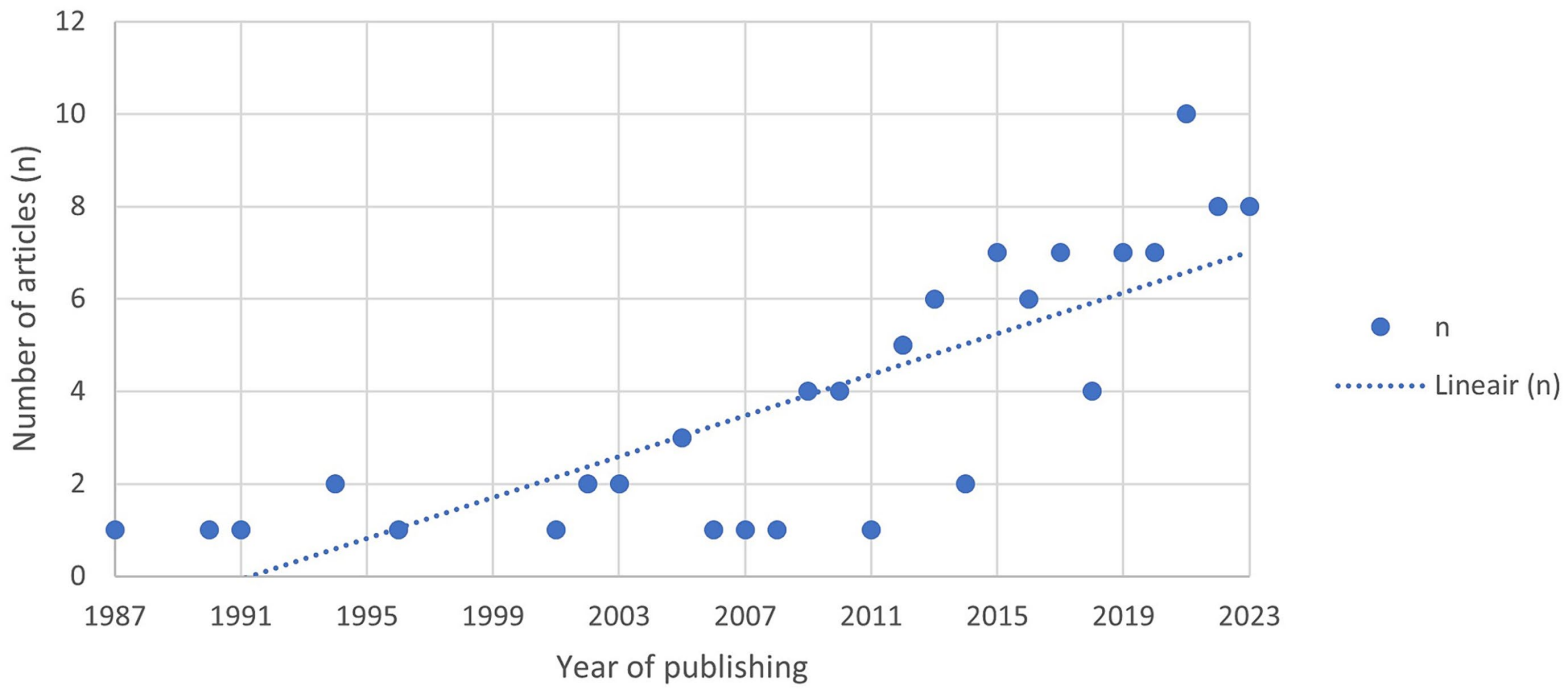
Overview of studies included in this review for analysis based on the in-and exclusion criteria, selected by year of publishing.

Following the search strategy 103 papers were included for further data analysis. The studies are listed in Supplementary Document 3. Figure 3 shows the total number of papers in our study, arranged by year of publication. The last decade showed a significant increase in SBVM papers, with 70% of the papers published in the last decade (*n* = 72 of *n* = 103 total papers).

The (sub) components of “Learning goals,” “Learning activities,” and “Learning outcomes” per study were succinctly summarized in Table 2.

### Learning goals

The “Learning goals” were divided into three components, “Relevant knowledge,” “Clinical reasoning,” and “Procedural steps.” 64% of the scrutinized studies focused on the acquisition of procedural steps.

### Learning activities

We have identified the simulator characteristics for the subcomponents: “Simulator technology,” “Type of simulator,” and “Use of simulator.” Notably, the simulator technology reveals an emphasis on static simulators (49%) and screen-based simulators (31%). These technologies are often classified as low-fidelity simulators due to their low level of complexity and interactivity. For type of simulator, part-task-driven simulators were two times more prevalent than their event-driven counterparts. For use of simulator, we have identified the majority of simulators as interactive in use (94%), only approximately one in 11 simulators was identified as non-interactive in use.

### Learning outcomes

The components for “Learning outcomes” were, “Knowledge,” “Application of knowledge,” “Competence,” and “Clinical performance.” The evaluated learning outcomes that were most identified were competence (58%) and knowledge (32%). In 70% of the studies self-evaluated learning outcomes were identified. Within the self-evaluated learning outcomes, a similar distribution is noted in comparison with evaluated Learning outcomes, except for the prevalence of clinical performance, which is notably more identified as a self-evaluated learning outcome.

As a next step we evaluated the relation between these categories.

### Learning outcomes in relation to learning goals

This analysis addresses the question of how “Learning outcomes” interact with the “Learning goals” of clinical skills training and gain insight in how they are aligned. Here we identify which learning outcomes are most frequently identified according to our described classification.

Multiple “Learning outcomes” could be identified within a single study. The learning outcomes were analyzed in relation to the primary “Learning goal,” aiming to demonstrate which learning outcomes were used to measure a specific learning goal of a clinical skill (Table 3). The percentages for each learning outcome are presented relative to all included studies, therefore resulting in a total exceeding 100%. In the context of acquiring “Relevant knowledge” and “Clinical reasoning” skills, “Knowledge” (94 and 78%) and “Application of knowledge” (28 and 72%) were primarily used as evaluated learning outcomes; and to a lesser extent in training of “Procedural steps” (6 and 2%). For the training of procedural steps, “Competence” was mostly (95%) used as a learning outcome. “Clinical performance” was more rarely reported and exclusively used as a learning outcome in a learning process with procedural steps as learning goal (6%).

**Table 3:** Overview of “Learning outcomes” related to distinct “Learning goals.”

Primary learning goal per study	Learning outcomes^a^^,^^b^			
	Knowledge [Self-eva]	Application of knowledge [Self-eva]	Competence [Self-eva]	Clinical performance [Self-eva]
Relevant knowledge	94% [73%]	28% [33%]	17% [40%]	0% [7%]
Clinical reasoning	78% [47%]	72% [60%]	6% [20%]	0% [20%]
Procedural steps	6% [26%]	2% [9%]	95% [98%]	6% [35%]

The percentages reflect the distribution of learning outcomes per category of a learning goals and show how different learning goals are evaluated in terms of evaluated learning outcomes and self-evaluated [Self-eva] learning outcomes (between brackets).

a For the analysis of evaluated learning outcomes only studies with one or multiple identified evaluated learning outcome are included. For the analysis of self-evaluated learning outcomes only studies with one or multiple identified self-evaluated learning outcome are included.

b Multiple learning outcomes could be identified within a single study. The percentages are relative to all included studies, therefore resulting in a total exceeding 100%.

The self-evaluated learning outcomes show a similar pattern. However, the results indicate that self-evaluated learning outcomes are more evenly used across all learning goals. For instance, the self-evaluation of clinical performance is notably more prevalent across all learning goals compared to evaluated learning outcomes.

The analysis further explored how different simulator characteristics were related to each type of “Learning goal” and “Learning outcome,” according to our systematic approach (as detailed in Tables 4A, 4B).

**Table 4A:** Overview of the percentage distribution of “Learning goals” with respect to simulator characteristics in the “Learning activity.”

Learning goals i.r.t.	Simulator characteristics in Learning activities^a^										
	Simulator technology						Type of simulator			Use of simulator	
	Static	Screen-based	Box trainer	Animal-derived	Integrated	XR	Part-task	Event	Hybrid	Interactive	Non-interactive
Relevant knowledge	25%	85%	0%	0%	0%	10%	20%	95%	5%	100%	20%
Clinical reasoning	26%	74%	0%	0%	0%	5%	26%	74%	5%	84%	21%
Procedural steps	74%	7%	20%	9%	9%	4%	110%	4%	9%	120%	3%

The percentages represent the prevalence of each simulator characteristic within specific learning goals category.

a Per study multiple simulators can be identified with accompanying characteristics and therefore the total can exceed 100%.

**Table 4B:** Overview of the distribution of “Learning outcomes” with respect to simulator characteristics in the “Learning activity.”

Learning outcomes^a^ i.r.t.	Simulator characteristics in Learning activities^b^										
	Simulator technology						Type of simulator			Use of simulator	
	Static	Screen-based	Box trainer	Animal-derived	Integrated	XR	Part-task	Event	Hybrid	Interactive	Non-interactive
Knowledge [Self-eva]	26% [60%]	77% [50%]	0% [7%]	0% [7%]	3% [0%]	6% [3%]	20% [67%]	83% [57%]	9% [3%]	91% [113%]	20% [40%]
Application of knowledge [Self-eva]	21% [28%]	79% [67%]	0% [0%]	0% [6%]	5% [11%]	5% [6%]	26% [33%]	79% [57%]	5% [3%]	89% [111%]	21% [11%]
Competence [Self-eva]	76% [67%]	14% [20%]	16% [15%]	10% [7%]	5% [7%]	8% [6%]	111% [98%]	13% [17%]	5% [7%]	124% [111%]	5% [6%]
Clinical performance [Self-eva]	25% [35%]	0% [20%]	25% [25%]	0% [15%]	50% [15%]	0% [10%]	50% [85%]	0% [20%]	50% [15%]	100% [115%]	0% [5%]

The percentages indicate the prevalence of each simulator characteristic within specific learning outcome categories: evaluated learning outcomes and self-evaluated (Self-eva) learning outcomes (between brackets).

a For the analysis of evaluated learning outcomes only studies with identified evaluated learning outcome are included. For the analysis of self-evaluated [Self-eva] learning outcomes only studies that with identified self-evaluated learning outcome are included.

b Per study multiple simulators can be identified with accompanying characteristics and therefore the total can exceed over 100%.

### Learning goals in relation to simulator characteristics

In the context of clinical skills training, specific simulator characteristics were predominantly identified for the different “Learning goals,” as categorized in this review. For “Relevant knowledge” and “Clinical reasoning” as primary learning goals, screen-based simulators (85 and 74%), event-driven simulators (95 and 74%), and interactive simulators (100 and 84%) were the most common characteristics that were identified. For the training of “Procedural steps,” the use of static simulators (74%), part-task-driven simulators (110%), and interactive simulators (120%) were mostly described (Table 4A).

### Learning outcomes in relation to simulator characteristics

In this section we describe how simulator characteristics in the learning activity relate to the measured learning outcomes in the clinical skills training setting. For the “Knowledge” and “Application of knowledge” learning outcomes, we identified certain simulator characteristics more frequently. Specifically, screen-based simulators, which display visual information on a screen, were identified in 77% (knowledge) and 79% (application of knowledge) of studies. Event-driven simulators, which are focused on a clinical scenario, were identified in 83 and 79% of the studies. Interactive simulators, which allow users to engage actively with the simulation, were the most frequently identified type, noted in 91 and 89% of the studies. In contrast, when analyzing “Competence” learning outcomes, which focus on practical skills and abilities, a different set of simulator characteristics was prominent. Specifically, static simulators, which do not change during use, were identified in 76% of studies. Part-task-driven simulators, which focus on specific parts of a task, were noted in 111% of studies. Interactive simulators were identified most, in 124% of the studies. For “Clinical performance” the analysis showed that integrated technologies (50%) and hybrid simulators (50%) were identified as learning outcomes in about half of all studies. Interactive simulators were identified in all studies (100%; Table 4B). However, it is important to note that clinical performance outcomes had limited representation in the dataset.

Several “Simulator technologies” are underrepresented in literature and therefore do not show clear relations when learning outcomes are positioned against simulator characteristics. The analysis showed only a strong connection between “Competence” outcomes and static simulators (76%; see Table 4B). In contrast, when reversed, meaning that when simulator characteristics are positioned against learning outcomes, the results indicated that, except for screen-based simulators, all types of simulator technologies were mainly associated with competence learning outcomes as well (see Table 5).

**Table 5:** Overview of simulator characteristics in relation to “Learning outcomes.”

	Percentage per category of simulator characteristic (%)										
Simulator characteristic i.r.t. Learning outcomes^a^^,^^b^	Simulator characteristics in learning activities										
	Simulator technology						Type of simulator			Use of simulator	
	Static	Screen-based	Box trainer	Animal-derived	Integrated	XR	Part-task	Event	Hybrid	Interactive	Non-interactive
Knowledge [Self-eva]	15% [44%]	75% [54%]	0% [25%]	0% [50%]	17% [0%]	33% [20%]	8% [36%]	83% [59%]	38% [17%]	27% [40%]	70% [67%]
Application of knowledge [Self-eva]	6% [12%]	42% [43%]	0% [0%]	0% [25%]	17% [40%]	17% [40%]	6% [11%]	43% [48%]	13% [33%]	14% [24%]	40% [33%]
Competence [Self-eva]	77% [88%]	25% [39%]	71% [100%]	100% [100%]	50% [80%]	83% [60%]	81% [95%]	23% [31%]	38% [67%]	65% [75%]	30% [50%]
Clinical performance [Self-eva]	2% [17%]	0% [14%]	7% [63%]	0% [75%]	33% [60%]	0% [40%]	2% [30%]	0% [14%]	25% [50%]	3% [27%]	0% [17%]

The percentages represent the prevalence of specific learning outcomes (self-evaluated [Self-eva] learning outcomes between brackets) within each simulator characteristic category.

a For the analysis of evaluated learning outcomes only studies with one or multiple identified evaluated learning outcome are included. For the analysis of self-evaluated [Self-eva] Learning outcomes only studies with one or multiple identified self-evaluated learning outcome are included.

b Multiple Learning outcomes could be identified per study, therefore the total may exceed over 100%.

When looking at “Self-evaluated learning outcomes,” where students evaluate their own learning, different patterns emerged. For self-evaluating “Knowledge” learning outcomes, the characteristics of simulators used were varied. Static simulators were identified in 60% of these studies, and screen-based simulators were identified in 50%. Both part-task-driven (67%) and event-driven (57%) simulators were most prevalent in the studies. Similar patterns were observed for self-evaluating “Application of knowledge” and “Competence” outcomes, although the differences between the types of simulators were less pronounced. Notably however, “Clinical performance” was more frequently identified as a self-evaluation learning outcome, with multiple simulator characteristics involved (Table 4B).

### Links between learning goals, learning activities, and learning outcomes

While individual studies might have explored the relationship between learning goals and learning outcomes, or looked at simulator characteristics, none of the studies in our dataset have combined all these elements to analyze how learning goals and learning outcomes are related to the characteristics of the simulator-based learning activities. In Tables 3, 4A, 4B the relationships between these (sub) components are summarized. For instance, the type of learning outcome “Competence” was related to the learning goal “Procedural steps” in 95% of the cases (Table 3). Competence and procedural steps, respectively, were associated with static simulator (76 and 74%), part-task-driven simulator (111 and 110%), and interactive simulators (124 and 120%; Tables 4A, 4B).

Furthermore, it was demonstrated that the type of learning outcome “Application of knowledge” was mostly associated with “Clinical reasoning” training (72%; Table 3). Simulator characteristics that were identified for a corresponding learning activity were, screen-based (79 and 74%), event-driven (79 and 74%), and interactive simulators (89 and 84%; Tables 4A, 4B).

## Discussion

This study investigated the interplay between “Learning goals,” “Learning activities,” and “Learning outcomes,” highlighting which simulator characteristics were used to create learning activities that align with both the learning goals” and the intended learning outcomes. As the literature indicates, in simulation-based education it is important to provide a structured, constructively aligned process where students experience relevant and effective education (7, 31–33). Through constructive alignment, students are more likely to understand what is expected from them, engage more effectively in activities that directly support their learning, and be assessed more accurately in a manner that reflects the intended learning outcomes (34–37). The result of this study underscores the importance of aligning the learning process in simulation-based clinical skills training.

Within the included studies we have identified a variety of simulator technology in SBVE. This study shows that static and screen-based simulators are most commonly identified. Static simulators were linked to the acquisition of “Procedural steps” and screen-based simulators were mostly linked to both “Relevant knowledge” and “Clinical reasoning” acquisition. Although our results were derived from the included studies, other publications outside the scope of our review have noted some limitations with respect to screen-based and static simulators (2, 38, 39). For example, Liebig et al. demonstrated in their study that screen-based simulators can deliver theoretical concepts effectively (40) but their ability to facilitate practical application may be questionable (15). This is supported by the study of Datta et al. which shows that the translation into real-world clinical skills of knowledge gained through the use of these simulators might not always be seamless (15). The study of Al-Elq described that static simulators are restricted in addressing the dynamic and unpredictable nature of real clinical environments, therefore limiting students in preparing for more complex clinical scenarios (14). In addition, screen-based and static simulators are considered more passive simulators through the lack of physical interaction and tactile feedback, which could hinder the development of crucial skills. These simulators do not actively support learning, and information obtained through active engagement is retained better than information acquired through passive education (15, 41). Thus, these findings highlight the importance of aligning “Simulator technology” with “Learning goals” and “Learning outcomes.”

In addition, other simulator characteristics were examined, such as “Type of simulator” and “Use of simulator.” This study demonstrates that part-task-driven simulators were linked to “Competence” learning outcomes. This is explained in the study of Datta et al. that reported part-task-driven simulators for their targeted skill practice and the ability to isolate and focus on specific aspects of a procedure. These simulators provide students with a safe environment, in which they can make and experience mistakes, to acquire and refine procedural skills before applying them in clinical settings (15). Furthermore, this systematic review reveals that event-driven simulators were primarily linked to learning outcomes regarding “Knowledge.” As reported by the study of Datta et al., event-driven simulators can excel in assessing knowledge-based learning outcomes, as they create contextualized scenarios for practical knowledge application (15).

Our study also investigated interactivity as a simulator characteristic. Most studies included in this review describe interactivity as an essential feature. This is supported by the papers of Musa et al. and Koukourikos et al. who reported that interactive simulators facilitate student engagement and promote active participation, enhancing skill acquisition (28, 42).

Understanding and addressing any misalignments within the learning process certainly helps to improve the overall educational experience and support students in achieving their learning goals (43, 44). These misalignments can occur not only in “Self-evaluated learning outcomes” but also in the instructional design and implementation of simulation-based education. By considering both the development and implementation perspectives, as well as the student’s interaction with the simulator, a more cohesive and effective learning environment is needed. Self-evaluated learning outcomes can give relevant insights into the quality of education in particular related to student’s self-confidence (40, 45). When students engage in self-evaluation, they assess their own learning progress, which can include aspects related to self-efficacy. Encouraging reflection and self-evaluation as part of the learning process can enhance self-efficacy and confidence. When students recognize their progress and acknowledge their accomplishments through reflective cycles, they are more likely to feel competent and motivated to continue learning. This approach corresponds with the principles of the experiential learning cycle, which emphasizes the importance of concrete experience, reflective observation, abstract conceptualization, and active experimentation. Bajpai et al. and Liew et al. described the importance of considering the diversity within a group of students in terms of “Self-evaluated learning outcomes” (44, 45). Students may have varying levels of confidence in their abilities, different goals they wish to achieve, and diverse ways of evaluating their own progress. This can influence how students perceive the quality of SBVE in the context of their own development. On the contrary, misalignment in the SBVE learning process, a disconnect between student expectations and actual educational outcomes, can result in students feeling less confident or satisfied with their learning experiences (40, 44, 45).

An example of where students may feel disengaged and less satisfied, is found in the dimensionality used in the learning process. The prevalence of two-dimensional (2D) simulators, such as screen-based ones, were frequently identified in the included studies but contrasts with the hands-on nature of training, as noted by Datta et al. and Liebig et al. (15, 40). Individual differences in spatial abilities further exacerbate this misalignment, particularly in learning anatomical knowledge. In clinical settings, understanding three-dimensional (3D) spatial relationships is important, yet both 2D and 3D simulators are used for training anatomical knowledge, despite their differing effectiveness (46–48). Moreover, measurement tools, mainly written exams but also, e.g., within-simulator tools to provide users with feedback, remain predominantly 2D. For effective learning, both the “Learning activity” and measurement tool should align with the “Learning goal,” emphasizing the necessity for 3D offerings (49). These findings show that the simulator characteristics play a crucial role in alignment of the learning process. Therefore, a one-size-fits-all approach to simulation-based learning can never apply for all students and stages of the curriculum (50). With its adaptable simulator characteristics, SBVE can provide educational experiences that can be tailored to meet the diverse needs of students. Further research is needed to explore how individual preferences and differences influence simulation-based clinical skills training.

It is of utmost importance to continue innovating educational methodologies and technologies that are proven to be beneficial for students and that can safeguard animal welfare. This underscores the need for educational institutions to embrace such innovations, ensuring that the training provided is both ethical and effective.

## Limitations

The limitations of this paper include those from the inclusion and exclusion criteria, and potential publication bias. Publication bias, a significant concern in systematic reviews, arises when studies with positive results are more likely to be included, while non-significant findings are excluded. Contributing factors include the inclusion of many small studies with positive outcomes, excluding non-English studies, and ignoring “grey literature” like conference proceedings and dissertations. Despite efforts to minimize publication bias, complete avoidance is unrealistic. Though “grey literature” was not automatically excluded, none adhered to the selection criteria.

The risk of bias assessment included the aspects of the risk of bias analysis for each study, according to the Cochrane guidelines. Most studies maintain a low risk of bias rating, demonstrating adherence to rigorous methodologies with respect to randomization, intervention knowledge, measurement consistency, and data analysis. It is important to note that the majority of the studies reported that outcome assessors were aware of the intervention, and it could potentially influence the assessment. However, due to the control measures generally mentioned, the overall bias remains low (Supplementary Figure 1).

A notable limitation of this study lies in the categorization process used to classify the reviewed papers. Although specific criteria were established for categorization, these criteria were developed by the reviewers and applied to the papers according to their assessments. Since the categories used in this framework were not explicitly defined in the original studies, the process involved making judgment calls to assign extracted data to the categories and subcomponents. This reliance on subjective interpretation introduces a potential for bias. The heterogeneity in research methodologies and approaches complicated the synthesis of findings, emphasizing the need for standardized evaluation metrics and methodologies in future studies. This variability highlights the evolving nature of SBVE research and the ongoing efforts to establish frameworks for assessing its impact on veterinary education.

## Conclusion

This study’s comprehensive analysis of “Learning goals,” “Learning activities,” and “Learning outcomes” within simulation-based veterinary education provides valuable insights for curriculum development in veterinary clinical skills training. Overall, the insights gained from this study have the potential to refine veterinary education by promoting evidence-based instructional strategies and the strategic integration of simulators. The study not only contributes to refining educational practices but also calls for a deeper consideration of the alignment between learning goals, learning activities, learning outcomes, and measurement tools. By embracing the potential of SBVE, educators can better cater to diverse student needs and foster optimized skill acquisition and application in veterinary clinical practice. By understanding the interconnectedness of learning goals, learning activities, learning outcomes, and measurement tools, educators could better foster skill acquisition and prepare students for real-world clinical scenarios with enhanced competence and self-efficacy.

## Data Availability

The original contributions presented in the study are included in the article/Supplementary material, further inquiries can be directed to the corresponding author.
