# Supplementary material for: Links between learning goals, learning activities, and learning outcomes in simulation-based clinical skills training: a systematic review of the veterinary literature
Source: Front Vet Sci. 2024 Oct 2;11:1463642. doi: 10.3389/fvets.2024.1463642 (PMC11479932; doi:10.3389/fvets.2024.1463642)
Supplement: Supplementary file 2 [file Table_1.docx]

Supplementary Document

Links between Learning Goals, Learning Activities, And Learning Outcomes In Simulation-based Clinical Skills Training: A Systematic Review Of The Veterinary Literature

**Neeltje J. Veenema*, Beerend P. Hierck, Harold G.J. Bok, Daniela C.F. Salvatori**

*** Correspondence:** n.j.veenema@uu.nl

# Supplementary Data

- 1. Systematic search

Database: Scopus
Search window: till June 2022
Date: July 4th 2022
( ( TITLE-ABS-KEY ( education  OR  training  OR  curriculum  OR  teaching  OR  learning ) )  AND  ( TITLE-ABS-KEY ( simulation  OR  simulations  OR  simulator  OR  model  OR  models ) )  AND  ( TITLE-ABS-KEY ( veterinary ) ) )  AND  ( TITLE-ABS-KEY ( outcomes  OR  effect  OR  effectiveness ) ) 
661 results

Database: Pubmed
Search window: till June 2022
Date: July 4th 2022
(("education"[Title/Abstract] OR "training"[Title/Abstract] OR "curriculum"[Title/Abstract] OR "teaching"[Title/Abstract] OR "learning"[Title/Abstract]) AND ("simulation"[Title/Abstract] OR "simulations"[Title/Abstract] OR "simulator"[Title/Abstract] OR "model"[Title/Abstract] OR "models"[Title/Abstract]) AND "veterinary"[Title/Abstract]) AND (outcomes[Title/Abstract] OR effect[Title/Abstract] OR effectiveness[Title/Abstract])
162 results

Database: Embase
Search window: till June 2022
Date: July 4th 2022
(education:kw,ab,ti OR training:kw,ab,ti OR curriculum:kw,ab,ti OR teaching:kw,ab,ti OR learning:kw,ab,ti) AND (simulation:kw,ab,ti OR simulations:kw,ab,ti OR simulator:kw,ab,ti OR model:kw,ab,ti OR models:kw,ab,ti) AND veterinary:kw,ab,ti AND (outcomes:ti,ab,kw OR effect:ti,ab,kw OR effectiveness:kw,ab,ti)
203 results

Database: ERIC
Search window: till June 2022
Date: July 4th 2022
(abstract:education OR abstract:training OR abstract:curriculum OR abstract:teaching OR abstract:learning) AND (abstract:simulation OR abstract:simulations OR abstract:simulator OR abstract:simulators OR abstract:model OR abstract:models) AND abstract:veterinary AND (abstract:outcomes OR abstract:effect OR abstract:effectiveness)
17 results

Database: CAB Abstracts
Search window: till June 2022
Date: July 4th 2022
(((ab:(outcomes) OR ab:(effect) OR ab:(effectiveness)) AND (ab:(veterinary)) AND (ab:(simulation) OR ab:(simulations) OR ab:(simulator) OR ab:(simulators) OR ab:(model) OR ab:(models)) AND (ab:(education) OR ab:(training) OR ab:(curriculum) OR ab:(teaching ) OR ab:(learning))))
136 results

Database: /. Manual searching
Date: till July 20th 2022
40 results

- 1. Screening results
     1. Piloting screening (Rayyan and ASReview)

Rayyan: finished July 18th
730 results
 581 results excluded based on title/abstract (obvious different topic).
 149 results on ‘maybe’ for now (related to topic).


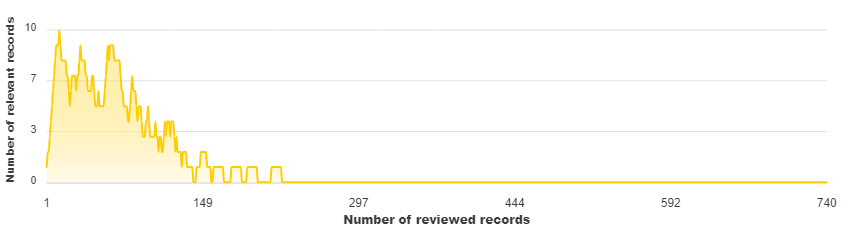
ASReview: finished July 21th
770 results
 677 irrelevant
 526 irrelevant after last relevant
 93 relevant results

- - 1. Formal screening (2nd reviewer)

Rayyan 770 results: finished July 26th
 665 results excluded based on title/abstract
 82 included results
 18 results were inconclusive; discussion with 2nd reviewer to reach consensus.

Total: 95 articles for full-text assessment for eligibility.
Total included articles: 91

- 1. Updated search strategy

Updated previous search strategy with search window: July 2022 till November 2023

New included articles: 12

Final number of articles in dataset for systemic review: 103
