# Supplementary material for: Links between learning goals, learning activities, and learning outcomes in simulation-based clinical skills training: a systematic review of the veterinary literature
Source: Front Vet Sci. 2024 Oct 2;11:1463642. doi: 10.3389/fvets.2024.1463642 (PMC11479932; doi:10.3389/fvets.2024.1463642)
Supplement: Supplementary file 4 [file Table_3.docx]

Supplementary Document

Links between Learning Goals, Learning Activities, And Learning Outcomes In Simulation-based Clinical Skills Training: A Systematic Review Of The Veterinary Literature

**Neeltje J. Veenema*, Beerend P. Hierck, Harold G.J. Bok, Daniela C.F. Salvatori**

*** Correspondence:** n.j.veenema@uu.nl

# Supplementary Data

Following the search strategy 103 articles were included for further data analysis.

1. Abutarbush SM, Naylor JM, Parchoma G, D'Eon M, Petrie L, Carruthers T. Evaluation of traditional instruction versus a self-learning computer module in teaching veterinary students how to pass a nasogastric tube in the horse. J Vet Med Educ. 2006;33(3):447-54.

2. Al-Khalili SM, Coppoc GL. 2D and 3D stereoscopic videos used as pre-anatomy lab tools improve students' examination performance in a veterinary gross anatomy course. J Vet Med Educ. 2014;41(1):68-76.

3. Allavena RE, Schaffer-White AB, Long H, Alawneh JI. Technical Skills Training for Veterinary Students: A Comparison of Simulators and Video for Teaching Standardized Cardiac Dissection. J Vet Med Educ. 2017;44(4):620-31.

4. Anderson SL, Miller L, Gibbons P, Hunt JA, Roberson J, Raines JA, et al. Development and validation of a bovine castration model and rubric. Journal of Veterinary Medical Education. 2021;48(1):96-104.

5. Anderson LS, Olin SJ, Whittemore JC. Proficiency and Retention of Five Clinical Veterinary Skills Using Multipurpose Reusable Canine Manikins vs. Live Animals: Model Development and Validation. Journal of veterinary medical education. 2022:e20220103.

6. Andrade EF, Zaine Teixeira Debortoli G, Gomes Batista VL, Newton Bizetto Meira de Andrade J, Orl, o DR, et al. Learning perception of Veterinary students about cardiovascular physiology using a functional model. Journal of Biological Education. 2020.

7. Ann, ale A, Ann, ale CH, Fosgate GT, Holm DE. Training Method and Other Factors Affecting Student Accuracy in Bovine Pregnancy Diagnosis. J Vet Med Educ. 2018;45(2):224-31.

8. Ann, ale A, Scheepers E, Fosgate GT. The effect of an ovariohysterectomy model practice on surgicaltimes for final-yearveterinary students' first live-animal ovariohysterectomies. Journal of Veterinary Medical Education. 2020;47(1):44-55.

9. Aulmann M, März M, Burgener IA, Alef M, Otto S, Mülling CK. Development and Evaluation of Two Canine Low-Fidelity Simulation Models. Journal of veterinary medical education. 2015;42(2):151-60.

10. Baillie S, Mellor DJ, Brewster SA, Reid SW. Integrating a bovine rectal palpation simulator into an undergraduate veterinary curriculum. J Vet Med Educ. 2005;32(1):79-85.

11. Baillie S, Crossan A, Brewster SA, May SA, Mellor DJ. Evaluating an automated haptic simulator designed for veterinary students to learn bovine rectal palpation. Simul Healthc. 2010;5(5):261-6.

12. Baillie S, Christopher R, Catterall AJ, Kruydenberg A, Lawrenson K, Wonham K, et al. Comparison of a silicon skin pad and a tea towel as models for learning a simple interrupted suture. Journal of Veterinary Medical Education. 2020;47(4):516-22.

13. Beaulieu A, Nykamp S, Phillips J, Arroyo LG, Koenig J, zur Linden A. Development and Validation of a Three-Dimensional Printed Training Model to Teach Ultrasound-Guided Injections of the Cervical Articular Process Joints in Horses. Journal of Veterinary Medical Education. 2022;49(4):442-53.

14. Bossaert P, Leterme L, Caluwaerts T, Cools S, Hostens M, Kolkman I, et al. Teaching transrectal palpation of the internal genital organs in cattle. J Vet Med Educ. 2009;36(4):451-60.

15. Branck CE, et al. The Validation of an Interactive Videodisc as an Alternative to Traditional Teaching Techniques: Auscultation of the Heart. Educational Technology. 1987;27(3):16-22.

16. Canright A, Bescoby S, Dickson J. Evaluation of a 3D Computer Model of the Equine Paranasal Sinuses as a Tool for Veterinary Anatomy Education. Journal of veterinary medical education. 2022:e20210134.

17. Carpenter LG, Piermattei DL, Salman MD, Orton EC, Nelson AW, Smeak DD, et al. A comparison of surgical training with live anesthetized dogs and cadavers. Veterinary surgery : VS : the official journal of the American College of Veterinary Surgeons. 1991;20(6):373-8.

18. Chen CY, Ragle CA, Lencioni R, Fransson BA. Comparison of 2 training programs for basic laparoscopic skills and simulated surgery performance in veterinary students. Veterinary Surgery. 2017;46(8):1187-97.

19. Cosford K, Briere J, Ambros B, Beazley S, Cartwright C. Effect of instructional format on veterinary students' task performance and emotional state during a simulation-based canine endotracheal intubation laboratory: Handout versus video. Journal of Veterinary Medical Education. 2020;47(2):239-47.

20. Crossan A, Brewster S, Mellor D, Reid S. Evaluating Training Effects of HOPS. 2003.

21. da Costa BN, Sousa MG, Tanji FN, Ulanin M, Wolf M, Stedile STO. The Use of 3-D Models of Echocardiographic Imaging Planes for Teaching Echocardiography Techniques for Use in Dogs and Cats. ATLA Alternatives to Laboratory Animals. 2022;50(3):208-20.

22. da Silva DAF, Fern, es AA, Ventrone AE, Dias A, Silveira AMS, et al. The influence of low-fidelity simulator training on canine peripheral venous puncture procedure. Veterinary World. 2021;14(2):410-8.

23. da Silva LCBA, Sellera FP, Gargano RG, Rossetto TC, Gomes GB, Miyahira FT, et al. Preliminary study of a teaching model for ultrasound-guided peripheral nerve blockade and effects on the learning curve in veterinary anesthesia residents. Veterinary Anaesthesia and Analgesia. 2017;44(3):684-7.

24. Denwood M, Dale VH, Yam P. Development and evaluation of an online computer-aided learning (CAL) package to promote small-animal welfare. J Vet Med Educ. 2008;35(2):318-24.

25. Eichel JC, Korb W, Schlenker A, Bausch G, Brehm W, Delling U. Evaluation of a training model to teach veterinary students a technique for injecting the jugular vein in horses. J Vet Med Educ. 2013;40(3):288-95.

26. Ertel RL, Braae UC, Ngowi HA, Johansen MV. Assessment of a computer-based Taenia solium health education tool 'The Vicious Worm' on knowledge uptake among professionals and their attitudes towards the program. Acta Trop. 2017;165:240-5.

27. Fawver AL, Branch CE, Trentham L, Robertson BT, Beckett SD. A comparison of interactive videodisc instruction with live animal laboratories. Am J Physiol. 1990;259(6):S11-4.

28. Fletcher DJ, Militello R, Schoeffler GL, Rogers CL. Development and evaluation of a high-fidelity canine patient simulator for veterinary clinical training. Journal of Veterinary Medical Education. 2012;39(1):7-12.

29. Fox V, Sinclair C, Bolt DM, Lowe J, Weller R. Design and validation of a simulator for equine joint injections. J Vet Med Educ. 2013;40(2):152-7.

30. Fransson BA, Ragle CA. Assessment of laparoscopic skills before and after simulation training with a canine abdominal model. J Am Vet Med Assoc. 2010;236(10):1079-84.

31. Fransson BA, Ragle CA, Bryan ME. Effects of two training curricula on basic laparoscopic skills and surgical performance among veterinarians. Journal of the American Veterinary Medical Association. 2012;241(4):451-60.

32. Fransson BA, Ragle CA, Mickas MM, Martin KW, Karn KNL. Ability to Perform Laparoscopic Intra- and Extracorporeal Suture Ligations in a Live Canine Ovariectomy Model after Simulation Training. Journal of Veterinary Medical Education. 2023;50(3):305-13.

33. Gao R, Liu J, Jing S, Mao W, He P, Liu B, et al. Developing a 3D animation tool to improve veterinary undergraduate understanding of obstetrical problems in horses. Veterinary Record. 2020;187(9).

34. Giusto G, Comino F, ini M. Validation of an effective, easy-to-make hemostasis simulator. Journal of veterinary medical education. 2015;42(1):85-8.

35. Gookin J, McWhorter D, Vaden S, Posner L. Outcome assessment of a computer-animated model for learning about the regulation of glomerular filtration rate. Advances in physiology education. 2010;34:97-105.

36. Gookin JL, Foster DM, Harvey AM, McWhorter D. An animated model of reticulorumen motility. J Vet Med Educ. 2009;36(4):444-7; quiz 8-50.

37. Gopinath D, McGreevy PD, Zuber RM, Klupiec C, Baguley J, Barrs VR. Developments in undergraduate teaching of small-animal soft-tissue surgical skills at the University of Sydney. Journal of Veterinary Medical Education. 2012;39(1):21-9.

38. Guaraná JB, Aytaç G, Müller AF, Thompson J, Freitas SH, Lee UY, et al. Extended reality veterinary medicine case studies for diagnostic veterinary imaging instruction: Assessing student perceptions and examination performance. Anat Histol Embryol. 2023;52(1):101-14.

39. Gunning P, Smith A, Fox V, Bolt DM, Lowe J, Sinclair C, et al. Development and validation of an equine nerve block simulator to supplement practical skills training in undergraduate veterinary students. Vet Rec. 2013;172(17):450.

40. Haines JM, Wardrop KJ, Lindberg CJ, Carbonneau KJ, Ngwenyama TR, Martin LG. Development and assessment of a formal learning module to educate veterinary students in an intensive care unit about transfusion reactions. J Vet Emerg Crit Care (San Antonio). 2020;30(4):405-10.

41. Heuwieser W, Oltenacu PA, Mansfeld R, Johnson PJ. Computer Based Instruction is Effective to Teach Dairy Reproductive Management. Journal of Veterinary Medicine Series A. 1994;41(1):329-32.

42. Hobbs KJ, Johnson PJ, Scharf ME, Cross DT, Wallace LL. Use of a jugular vein model for the instruction of equine intravenous catheter placement by third-and fourth-year veterinary students. Equine Veterinary Education. 2021;33(5):249-54.

43. Hunt JA, Schmidt P, Perkins J, Newton G, Anderson SL. Educational Research Report Comparison of Three Canine Models for Teaching Veterinary Dental Cleaning. J Vet Med Educ. 2021;48(5):573-83.

44. Johnson MD, Behar-Horenstein LS, MacIver MA, Su Y. Assessing the Effectiveness of a Cadaveric Teaching Model for Performing Arthrocentesis with Veterinary Students. Journal of veterinary medical education. 2016;43(1):88-94.

45. Johnson KL, Hespel AM, Price JM, de Swarte M. Use of color-coded, three-dimensional-printed equine carpus models is preferred by students but does not result in statistically different academic performance. Veterinary Radiology and Ultrasound. 2021;62(1):76-83.

46. Jones JL, Rinehart J, Englar RE. The effect of simulationtraining in anesthesia on student operational performance and patient safety. Journal of Veterinary Medical Education. 2019;46(2):205-13.

47. Keegan R, Henderson T, Brown G. Use of the virtual ventilator, a screen-based computer simulation, to teach the principles of mechanical ventilation. Journal of veterinary medical education. 2009;36(4):436-43.

48. Keegan RD, Brown GR, Gordon A. Use of a simulation of the ventilator-patient interaction as an active learning exercise: Comparison with traditional lecture. Journal of Veterinary Medical Education. 2012;39(4):359-67.

49. Kerr CL, Abdulghani M, Smith C, Khosa DK. Randomized Trial Comparing Instructor-Delivered Feedback with Self-Assessment Using Video During Basic Skills Training. Journal of veterinary medical education. 2021:e20210123.

50. Khalil MK, Lamar CH, Johnson TE. Using computer-based interactive imagery strategies for designing instructional anatomy programs. Clin Anat. 2005;18(1):68-76.

51. Kleinsorgen C, von Köckritz-Blickwede M, Naim HY, Branitzki-Heinemann K, Kankofer M, Mándoki M, et al. Impact of Virtual Patients as Optional Learning Material in Veterinary Biochemistry Education. J Vet Med Educ. 2018;45(2):177-87.

52. Lee S, Lee J, Lee A, Park N, Lee S, Song S, et al. Augmented reality intravenous injection simulator based 3D medical imaging for veterinary medicine. The Veterinary Journal. 2013;196(2):197-202.

53. Leeuwen BSv, Dollé AED, Vernooij JCM, Hierck BP, Salvatori DCF. Rotation of 3D Anatomy Models Is Associated with Underperformance of Students with Low Visual-Spatial Abilities: A Two-Center Randomized Crossover Trial. Education Sciences. 2023;13(10):992.

54. Levi O, Michelotti K, Schmidt P, Lagman M, Fahie M, Griffon D. Comparison between Training Models to Teach Veterinary Medical Students Basic Laparoscopic Surgery Skills. Journal of veterinary medical education. 2016;43(1):80-7.

55. Levi O, Shettko DL, Battles M, Schmidt PL, Fahie MA, Griffon DJ, et al. Effect of short- Versus long-term video game playing on basic laparoscopic skills acquisition of veterinary medicine students. Journal of Veterinary Medical Education. 2019;46(2):184-94.

56. Linton A, Garrett AC, Ivie KR, Jones JD, Martin JF, Delcambre JJ, et al. Enhancing Anatomical Instruction: Impact of a Virtual Canine Anatomy Program on Student Outcomes. Anatomical sciences education. 2022;15(2):330-40.

57. Linton A, Schoenfeld-Tacher R, Whalen LR. Developing and implementing an assessment method to evaluate a virtual canine anatomy program. J Vet Med Educ. 2005;32(2):249-54.

58. Little WB, Artemiou E, Conan A, Sparks C. Computer assisted learning: assessment of the veterinary virtual anatomy education software IVALA<sup>TM</sup>. Veterinary Sciences. 2018;5(2).

59. Luís Pires J, Payo P, Marcos R. The Use of Simulators for Teaching Fine Needle Aspiration Cytology in Veterinary Medicine. Journal of veterinary medical education. 2022;49(1):39-44.

60. Lumbis RH, Gregory SP, Baillie S. Evaluation of a dental model for training veterinary students. Journal of Veterinary Medical Education. 2012;39(2):128-35.

61. MacArthur SL, Johnson MD, Colee JC. Effect of a spay simulator on student competence and anxiety. Journal of Veterinary Medical Education. 2021;48(1):115-27.

62. Marcos R, Fonte-Oliveira L, Santos M, Caniatti M. An immersive simulation strategy to teach cytology sample collection methods and basic diagnosis skills: A two academic center study. Veterinary Clinical Pathology. 2023.

63. Marcos R, Macedo S, de Vega M, Payo-Puente P. The Use of Simulation Models and Student-Owned Animals for Teaching Clinical Examination Procedures in Veterinary Medicine. Veterinary Sciences. 2023;10(3).

64. McCool KE, Bissett SA, Hill TL, Degernes LA, Hawkins EC. Evaluation of a humanvirtual-reality endoscopytrainer forteaching early endoscopy skills toveterinarians. Journal of Veterinary Medical Education. 2020;47(1):106-16.

65. Modell JH, Cantwell S, Hardcastle J, Robertson S, Pablo L. Using the human patient simulator to educate students of veterinary medicine. J Vet Med Educ. 2002;29(2):111-6.

66. Nagel C, Ille N, Aurich J, Aurich C. Teaching of diagnostic skills in equine gynecology: simulator-based training versus schooling on live horses. Theriogenology. 2015;84(7):1088-95.

67. Nemanic S, Mills S, Viehdorfer M, Clark T, Bailey M. The effectiveness of a 3D computerized tutorial to enhance learning of the canine larynx and hyoid apparatus. Journal of Veterinary Medical Education. 2016;43(3):243-54.

68. Nibblett BMD, Pereira MM, Williamson JA, Sithole F. Validation of a model for teaching canine Fundoscopy. Journal of Veterinary Medical Education. 2015;42(2):133-9.

69. Nibblett BMD, Pereira MM, Sithole F, Orchard PAD, Bauman EB. Design and validation of a three-dimensional printed flexible canine otoscopy teaching model. Simulation in Healthcare. 2017;12(2):91-5.

70. Noyes JA, Keegan RD, Carbonneau KJ, Lepiz ML, Rankin DC, Matthew SM. Evaluating a Multimodal Clinical Anesthesia Course Integrated Into an Existing Veterinary Curriculum. Simulation in healthcare : journal of the Society for Simulation in Healthcare. 2021;16(3):177-84.

71. Olsen D, Bauer MS, Seim HB, Salman MD. Evaluation of a hemostasis model for teaching basic surgical skills. Veterinary surgery : VS : the official journal of the American College of Veterinary Surgeons. 1996;25(1):49-58.

72. Onuk B, Çolak A, Arslan S, Sızer SS, Kabak M. The effects of clay modeling and plastic model dressing techniques on veterinary anatomy training. Kafkas Universitesi Veteriner Fakultesi Dergisi. 2019;25(4):545-51.

73. Pardo MA, Sumner JP, Friello A, Fletcher DJ, Goggs R. Assessment of the percutaneous dilatational tracheostomy technique in experimental manikins and canine cadavers. Journal of Veterinary Emergency and Critical Care. 2019;29(5):484-94.

74. Perez-Rivero JJ, Rendón-Franco E. Validation of the educational potential of a simulator to develop abilities and skills for the creation and maintenance of an intravenous cannula. ATLA Alternatives to Laboratory Animals. 2011;39(3):257-60.

75. Pérez-Rivero JJ, Batalla-Vera T, Rendón-Franco E. Development and validation of a low-fidelity simulator to suture a laparotomy in rabbits. Altern Lab Anim. 2015;43(4):P44-8.

76. Perez-Rivero JJ, Barbosa-Callejas IM, Delgado-Garduno L, Rodriguez-Buitron L, Lavalle-Avalos AE, Herrera-Barragan JA. A low-fidelity simulator for the development of vascular ligation skills. ATLA, Alternatives to Laboratory Animals. 2022;50(3):195-200.

77. Pinckney RD, Mealy MJ, Thomas CB, MacWilliams PS. Impact of a computer-based auto-tutorial program on parasitology test scores of four consecutive classes of veterinary medical students. J Vet Med Educ. 2001;28(3):136-9.

78. Popa C, Pestean C, Vladut G, Vranceanu DS, Panaitescu PS, Al-Hajjar N, et al. Basic laparoscopic training program for veterinary medicine students: first Romanian pilot study. Revista Romana de Medicina Veterinara. 2019;29(3):21-8.

79. Preast V, Danielson J, Bender H, Bousson M. Effectiveness of a computer-based tutorial for teaching how to make a blood smear. Vet Clin Pathol. 2007;36(3):245-52.

80. Preece D, Williams SB, Lam R, Weller R. "Let's get physical": advantages of a physical model over 3D computer models and textbooks in learning imaging anatomy. Anat Sci Educ. 2013;6(4):216-24.

81. Rawson RE, Quinlan KM. Evaluation of a computer-based approach to teaching acid/base physiology. Adv Physiol Educ. 2002;26(1):85-97.

82. Rawson RE, Dispensa ME, Goldstein RE, Nicholson KW, Vidal NK. A simulation for teaching the basic and clinical science of fluid therapy. Adv Physiol Educ. 2009;33(3):202-8.

83. Read EK, Vallev, A, Farrell RM. Evaluation of Veterinary Student Surgical Skills Preparation for Ovariohysterectomy Using Simulators: A Pilot Study. J Vet Med Educ. 2016;43(2):190-213.

84. Reiter R, Viehdorfer M, Hescock K, Clark T, Nemanic S. Effectiveness of a Radiographic Anatomy Software Application for Enhancing Learning of Veterinary Radiographic Anatomy. J Vet Med Educ. 2018;45(1):131-9.

85. Romero C, Mendoza G, Martínez JA, Hernández PA, Magallon E, García-Contreras A. Evaluation of psychomotor skills acquired for surgery by veterinary students using biological simulators. Interciencia. 2013;38:377-81.

86. Rousseau M, Beauchamp G, Nichols S. Evaluation of a jugular venipuncture alpaca model to teach the technique of blood sampling in adult alpacas. Journal of Veterinary Medical Education. 2017;44(4):603-11.

87. Scherzer J, Buchanan MF, Moore JN, White SL. Teaching veterinary obstetrics using three-dimensional animation technology. J Vet Med Educ. 2010;37(3):299-303.

88. Schlesinger SL, Dahlberg M, Heuwieser W, Fischer-Tenhagen C. Examining the Role of Structured Debriefing in Simulator-Based Clinical Skills Training for Namibian Veterinary Students: A Pilot Study. Journal of veterinary medical education. 2021;48(6):656-63.

89. Schlesinger SL, Heuwieser W, Fischer-Tenhagen C. Using an OSCE to Explore the Role of Structured Debriefing and Self-Directed Learning in Simulator-Based Clinical Skill Training in Production Animal Reproductive Medicine. J Vet Med Educ. 2022:e20210060.

90. Schoenfeld-Tacher RM, Horn TJ, Scheviak TA, Royal KD, Hudson LC. Evaluation of 3D additively manufactured canine brain models for teaching veterinary neuroanatomy. Journal of Veterinary Medical Education. 2017;44(4):612-9.

91. Schoenfeld-Tacher RM, McConnell SL, Schultheiss T. Use of interactive online histopathology modules at different stages of a veterinary program. J Vet Med Educ. 2003;30(4):364-71.

92. Seguino A, Seguino F, Eleuteri A, Rhind SM. Development and evaluation of a virtual slaughterhouse simulator for training and educating veterinary students. J Vet Med Educ. 2014;41(3):233-42.

93. Silveira EEd, Silva Lisboa Neto AFd, Pereira HCS, Ferreira JS, Santos ACd, Siviero F, et al. Canine skull digitalization and three-dimensional printing as an educational tool for anatomical study. Journal of Veterinary Medical Education. 2021;48(6):649-55.

94. Smeak DD, Hill LN, Beck ML, Shaffer CA, Birchard SJ. Evaluation of an autotutorial-simulator program for instruction of hollow organ closure. Vet Surg. 1994;23(6):519-28.

95. Suñol A, Aige V, Morales C, López-Beltran M, Lujan-Feliu-Pascual A, ro, et al. Use of Three-Dimensional Printing Models for Veterinary Medical Education: Impact on Learning How to Identify Canine Vertebral Fractures. Journal of Veterinary Medical Education. 2019;46:1-10.

96. Tapia-Araya AE, Usón-Gargallo J, Enciso S, Pérez-Duarte FJ, Díaz-Güemes Martin-Portugués I, Fresno-Bermejo L, et al. Assessment of Laparoscopic Skills in Veterinarians Using a Canine Laparoscopic Simulator. J Vet Med Educ.43(1):71-9.

97. Thompson JL, MacKay J, Bowlt Blacklock K. Evaluation of veterinary students’ confidence and competence with surgical entrustable professional activities after repeated use of low-fidelity training models. Veterinary Record. 2023;192(8):no.

98. Troy JR, Bergh MS. Development and Efficacy of a Canine Pelvic Limb Model Used to Teach the Cranial Drawer and Tibial Compression Tests in the Stifle Joint. Journal of veterinary medical education. 2015;42(2):127-32.

99. Walton R, Riha J, Swor T, Kopper J, Yuan L, Mochel J, et al. Comparison of Traditional Didactic vs. Additional Hands-on Simulation Training in the Performance of Basic Life Support in Veterinary Students - A Prospective, Blinded, Randomized Study. J Vet Med Educ. 2023:e20220121.

100. Williamson JA, Hecker K, Yvorchuk K, Artemiou E, French H, Fuentealba C. Development and validation of a feline abdominal palpation model and scoring rubric. The Veterinary record. 2015;177(6):151.

101. Williamson JA, Dascanio JJ, Christmann U, Johnson JW, Rohleder B, Titus L. Development and Validation of a Model for Training Equine Phlebotomy and Intramuscular Injection Skills. Journal of veterinary medical education. 2016;43(3):235-42.

102. Williamson JA, Brisson BA, Anderson SL, Farrell RM, Spangler D. Comparison of 2 canine celiotomy closure models for training novice veterinary students. Veterinary surgery : VS. 2019;48(6):966-74.

103. Zambelli D, Canova M, Ballotta G, Ferrari A, Cunto M. Innovative models for teaching reproduction in small animals: The experience at DIMEVET of Bologna University. Theriogenology. 2023;196:244-53.
