# Supplementary material for: Links between learning goals, learning activities, and learning outcomes in simulation-based clinical skills training: a systematic review of the veterinary literature
Source: Front Vet Sci. 2024 Oct 2;11:1463642. doi: 10.3389/fvets.2024.1463642 (PMC11479932; doi:10.3389/fvets.2024.1463642)

Supplementary Material

Links between Learning Goals, Learning Activities, And Learning Outcomes In Simulation-based Clinical Skills Training: A Systematic Review Of The Veterinary Literature

**Neeltje J. Veenema*, Beerend P. Hierck, Harold G.J. Bok, Daniela C.F. Salvatori**

*** Correspondence:** n.j.veenema@uu.nl

## Supplementary Figures

Supplemanty Figure 1: separate JPEG file

Caption for Supplementary Figure 1:

**Supplementary Figure 1.** This figure presents the risk of bias assessment for each individual study included in the systematic review, using the Cochrane Risk of Bias Tool.


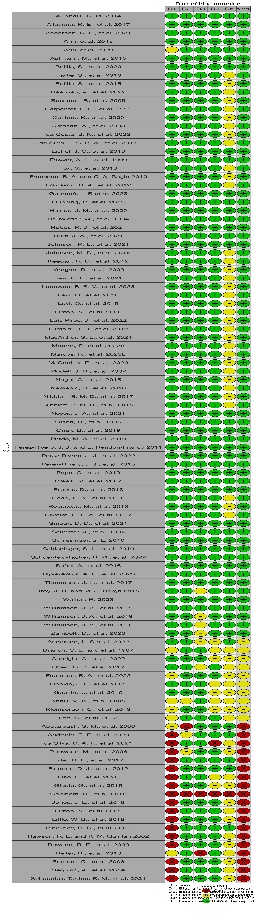

Supplement: Supplementary file 5 [file Table_4.docx]
